# Incidence and predictors of hospital readmission in children presenting with severe anaemia in Uganda and Malawi: a secondary analysis of TRACT trial data

**DOI:** 10.1186/s12889-021-11481-6

**Published:** 2021-07-29

**Authors:** Roisin Connon, Elizabeth C. George, Peter Olupot-Olupot, Sarah Kiguli, George Chagaluka, Florence Alaroker, Robert O. Opoka, Ayub Mpoya, Kevin Walsh, Charles Engoru, Julius Nteziyaremye, Macpherson Mallewa, Neil Kennedy, Margaret Nakuya, Cate Namayanja, Eva Nabawanuka, Tonny Sennyondo, Denis Amorut, C. Williams Musika, Imelda Bates, M. Boele van Hensbroek, Jennifer A. Evans, Sophie Uyoga, Thomas N. Williams, Gary Frost, Diana M. Gibb, Kathryn Maitland, A. Sarah Walker, on behalf of the TRACT trial group, S. Kiguli, R. O. Opoka, E. Nabawanuka, J. Kayaga, C. Williams Musika, E. Kadama, I. Mbwali, L. Nuwabaine, R. Nakikwaku, J. Nsubuga, K. Mpande, R. Adoo, O. Ouma, N. K. Adia, P. Olupot-Olupot, J. Nteziyaremye, C. Namanyanga, G. Passi, T. Sennyondo, R. Adong, C. B. Okalebo, E. Atimango, S. Mwamula, J. Kapsindet, G. Kiluli R. Muhindo, G. Masifa N. Thembo, G. Odong, C. Engoru, F. Aloroker, M. Nakuya, D. Amorut, M. Ariima, M. Itipe, M. G. Atim, M. Abeno, B. Amede, M. Olupot, S. Okwi, M. G. Kulume, G. Among, P. Onyas, E. D. Achipa, K. Maitland, A. Mpoya, P. Maitha, S. Uyoga, T. N. Williams, A. Macharia, M. Mallewa, G. Chagaluka, Y. Chimalizeni, N. Kennedy, F. Kumwenda, E. Nkosi, T. Sochera, A. Malenga, B. Gushu, T. Phiri, A. Chisale, N. Mitole, E. Chokani, A. Munthali, K. Maitland, T. N. Williams, G. Frost, K. Walsheto, D. M. Gibb, E. C. George, M. Thomason, D. Baptiste, L. McCabe, A. S. Walker, A. Ali, K. Khamis, M. Madula, G. Abongo, R. Heydermann, I. Bates, B. Urban, M. Boele van Hensbroek, F. Kyomuhendo, S. Nakalanzi, J. Chabuka, N. Mkandawire, J. A. Evans, D. M. Gibb, F. Fitzgerald, E. Molyneux, I. Lubega M. Murphy, P. Kazembe, J. Crawley, T. Peto, P. Musoke, J. Todd, G. Mirembe, F. Tenu

**Affiliations:** 1 MRC Clinical Trials Unit at University College London, 90 High Holborn, London, WC1V 6LJ UK; 2 Mbale Clinical Research Institute, Pallisa Road, PO Box 291, Mbale, Uganda; 3 Faculty of Health Sciences, Busitema University, PO Box 236, Tororo, Uganda; 4 Department of Paediatrics and Child Health, School of Medicine, Makerere University and Mulago Hospital, PO Box 7072, Kampala, Uganda; 5 College of Medicine, and Malawi-Liverpool-Wellcome Trust Clinical Research Programme, Blantyre, Malawi; 6 Soroti Regional Referral Hospital, PO Box 289, Soroti, Uganda; 7 KEMRI-Wellcome Trust Research Programme, PO Box 230, Kilifi, Kenya; 8 Institute of Global Health and Innovation, Department of Medicine, Imperial College London, London, SW7 2AZ UK; 9 School of Medicine, Dentistry and Biomedical Science, Queen’s University Belfast, Belfast, UK; 10 Liverpool School of Tropical Medicine and Hygiene, Liverpool, UK; 11 Emma Children’s Hospital AMC Amsterdam, Amsterdam, The Netherlands; 12 Department of Paediatrics, University Hospital of Wales, Heath Park Cardiff, Cardiff, CF14 4XW Wales

**Keywords:** Severe anaemia, Readmission

## Abstract

**Background:**

Severe anaemia (haemoglobin < 6 g/dL) is a leading cause of recurrent hospitalisation in African children. We investigated predictors of readmission in children hospitalised with severe anaemia in the TRACT trial (ISRCTN84086586) in order to identify potential future interventions.

**Methods:**

Secondary analyses of the trial examined 3894 children from Uganda and Malawi surviving a hospital episode of severe anaemia. Predictors of all-cause readmission within 180 days of discharge were identified using multivariable regression with death as a competing risk. Groups of children with similar characteristics were identified using hierarchical clustering.

**Results:**

Of the 3894 survivors 682 (18%) were readmitted; 403 (10%) had ≥2 re-admissions over 180 days. Three main causes of readmission were identified: severe anaemia (*n* = 456), malaria (*n* = 252) and haemoglobinuria/dark urine syndrome (*n* = 165). Overall, factors increasing risk of readmission included HIV-infection (hazard ratio 2.48 (95% CI 1.63–3.78), *p* < 0.001); ≥2 hospital admissions in the preceding 12 months (1.44(1.19–1.74), *p* < 0.001); history of transfusion (1.48(1.13–1.93), *p* = 0.005); and missing ≥1 trial medication dose (proxy for care quality) (1.43 (1.21–1.69), *p* < 0.001). Children with uncomplicated severe anaemia (Hb 4-6 g/dL and no severity features), who never received a transfusion (per trial protocol) during the initial admission had a substantially lower risk of readmission (0.67(0.47–0.96), *p* = 0.04). Malaria (among children with no prior history of transfusion) (0.60(0.47–0.76), *p* < 0.001); younger-age (1.07 (1.03–1.10) per 1 year younger, *p* < 0.001) and known sickle cell disease (0.62(0.46–0.82), *p* = 0.001) also decreased risk of readmission. For anaemia re-admissions, gross splenomegaly and enlarged spleen increased risk by 1.73(1.23–2.44) and 1.46(1.18–1.82) respectively compared to no splenomegaly.

Clustering identified four groups of children with readmission rates from 14 to 20%. The cluster with the highest readmission rate was characterised by very low haemoglobin (mean 3.6 g/dL). Sickle Cell Disease (SCD) predominated in two clusters associated with chronic repeated admissions or severe, acute presentations in largely undiagnosed SCD. The final cluster had high rates of malaria (78%), severity signs and very low platelet count, consistent with acute severe malaria.

**Conclusions:**

Younger age, HIV infection and history of previous hospital admissions predicted increased risk of readmission. However, no obvious clinical factors for intervention were identified. As missing medication doses was highly predictive, attention to care related factors may be important.

**Trial registration:**

ISRCTN ISRCTN84086586.

**Supplementary Information:**

The online version contains supplementary material available at 10.1186/s12889-021-11481-6.

## Background

Severe anaemia (haemoglobin (Hb) < 6 g/dL) is a leading cause of hospitalisation and mortality in children in sub-Saharan Africa [1–4]. Guidelines developed by the World Health Organization (WHO) encourage the rational use of blood transfusion to preserve scarce resources of donor blood and to reduce the risk of transfusion-transmitted infections [5]. Specifically, they recommend not to transfuse children who have a haemoglobin between 4 and 6 g/dL without severity features. They also recommend 20 ml/kg of whole blood (or 10 ml/kg red cell concentrates) for all levels of anaemia below Hb < 6 g/dL [6]. However, if standard formulae are used to calculate volume required [7] this would result in under-treatment children with profound anaemia (haemoglobin < 4 g/dL) by ~ 30% as this volume is insufficient to correct anaemia [8].

The evidence base supporting these guidelines is weak, and as a result adherence is poor [8, 9]. A Cochrane review published in 2000 included the only 2 African randomised controlled trials (RCTs) [10, 11] conducted (involving 114 and 116 children randomised to blood transfusion or oral haematinics) concluded that there was insufficient information on whether routinely giving blood to clinically stable children with severe anaemia reduces death or improves haematological correction measured at 1 month, and indicated the need for a definitive trial [12]. A subsequent study reporting on a retrospective analysis of a multi-site observational study, including over 25,000 children hospitalised with severe malaria, reported an apparent benefit of blood transfusion on in-hospital survival [13]. However, the findings were challenged on the basis of selection bias since the analysis did not account for the confounding effect of time of death [14]. Death in children with severe malarial anaemia often occurs within hours of admission [15], including children awaiting a transfusion, as previously demonstrated [8].

The aetiology of severe anaemia is frequently multi-factorial, including potentially treatable co-morbidities such as malaria, bacteraemia and multiple vitamin deficiencies - key determinants of outcome [3] that are not addressed in current treatment guidelines. Outcomes remain unsatisfactory with high rates of reported in-hospital mortality in cohort studies (9–10%) [2, 3], and high additional mortality (8%), anaemia relapse (6%) and re-admission (17%) in the 6-months post-discharge [4]. The poor outcomes and recurrent morbidity of children with severe anaemia warranted a definitive trial to establish best transfusion and treatment strategies to prevent both early and delayed mortality and relapse. The TRACT trial (ISRCTN84086586) was designed as a multicentre trial, which enrolled 3983 children with severe anaemia from four centres in Uganda and Malawi, to investigate four different interventions to reduce the burden of morbidity/mortality. The specific hypotheses were (1) whether an immediate blood transfusion in children with uncomplicated severe anaemia (Hb 4-6 g/dl) would improve outcomes, (2) whether 30mls/kg versus 20mls/kg whole blood (or 15mls/kg vs 10mls/kg red cell concentrate) might improve outcomes, defined for both transfusion randomisations as mortality to day 28 (primary outcome) and day 180, and the need for additional transfusions and relapse of severe anaemia [16] and (3) whether multivitamin multimineral supplements (MVMM) using ‘Sprinkles’ formulation [17] vs iron and folate (standard of care) given for 3 months post-discharge and (4) whether infection prophylaxis cotrimoxazole for 3 months post-discharge vs no cotrimoxazole would reduce mortality and readmissions to day 28 (primary outcome) and day 180. The transfusion trial results, published in two linked manuscripts in 2019 [18, 19], demonstrated that an immediate transfusion is not required in children with uncomplicated severe anaemia (Hb 4-6 g/dL without severity features), as long as children are monitored for progression to the development of severe and/or complicated anaemia during their initial hospital stay; such progression occurred in 49% of participants in the control arm, who then received a transfusion triggered by the development of new signs of clinical severity or a drop in haemoglobin level to less than 4 g/dL [18]. The receipt of an immediate transfusion or no immediate transfusion did not predict longer term mortality or readmission to hospital. In children with severe anaemia who received a larger volume of blood, 30mls/kg whole blood equivalent (experimental), versus the 20 ml/kg (recommended in the current WHO guidelines) who did not have a fever (axillary temperature ≤ 37.5 °C, measured at screening) halved the number of deaths by 28 days (HR 0.43 (95%CI 0.27, 0.69)) [19]. Conversely, in children who were febrile (> 37.5 °C) at screening 30mls/kg whole blood equivalent almost doubled the risk of death in comparison to children who received the lower, 20 ml/kg, volume (HR 1.91 (95%CI 1.04, 3.49; *p*-value for heterogeneity of effect on mortality *p* = 0.00009). The micronutrient and infection prophylaxis trials were reported in a separate manuscript which demonstrated that neither enhanced supplementation with multivitamin multimineral versus iron and folate treatment nor co-trimoxazole prophylaxis improved 6-month survival or readmission to hospital [20].

One notable finding from the trial was the substantial rate of readmissions in the 6 months post-discharge, occurring in around 1 in 6 children. Here we investigate cause-specific incidence and predictors of hospital re-admissions in order to identify potential future interventions.

## Methods

### Study setting

The TRACT trial recruited children from Uganda (Mbale Regional Referral Hospital, Soroti Regional Hospital, and Mulago National Hospital, Kampala) and Malawi (Department/College of Medicine and Malawi-Liverpool-Wellcome Trust Clinical Research Programme, Blantyre). The sites were selected to represent a diversity of malaria transmission. In Eastern Uganda malaria endemicity is high and perennial (Mbale and Soroti centers); in Kampala (Mulago) Uganda malaria transmission is low; in Blantyre, in Southern Malawi malaria endemicity is moderate and highly seasonal. Enrolment began in September 2014 and completed in May 2017 when the full sample size was achieved. The last patient was followed up in November 2017.

### Original trial design

Eligible children were aged 2 months to 12 years presenting to hospital with severe anaemia (Hb < 6 g/dL); children with transfusions during the same admission, with known malignancy, trauma or surgery as main reason for admission, signs of bi-ventricular heart failure, chronic diseases or exclusively breast-fed infants were excluded [16]. The trial had two strata. Stratum A (complicated severe anaemia) included children with a haemoglobin < 6 g/dL with one or more severity features/symptoms (reduced conscious level (coma or prostration), respiratory distress, acute haemoglobinuria or known sickle cell disease (SCD)) or a haemoglobin < 4 g/dL (profound anaemia) irrespective of symptoms. Children in stratum A were given immediate transfusion randomised (ratio 1:1) to a volume of 30 or 20 mls/kg whole blood (15 vs 10 mls/kg red cell concentrates, denoted as whole blood equivalent). Stratum (TRACT) B (children with uncomplicated severe anaemia) included children with a Hb 4-6 g/dL with no severity features/symptoms who were randomised (1:1:2) to 30 or 20 mls/kg whole blood (15 vs 10 mls/kg red cell concentrates) or to no immediate transfusion (control: standard of care). Children in the control arm could receive a transfusion if the clinical severity criteria were met or Hb fell below 4 g/dL [16]. At the same time, all children were randomised using a factorial open-label design to receive 3 months post discharge cotrimoxazole prophylaxis or not, and to 3 months of MVMM supplementation (containing 10 mg iron as microencapsulated ferrous folate and 150 micrograms folic acid in addition to vitamins A, B1 (thiamine), B2 (riboflavin), B3 (niacin), B6, B12, C, D, zinc, copper, selenium and iodine given daily for all ages) versus standard treatment with iron (either 25 mg iron for children < 2 years or 60 mg iron for children ≥2 years) and folate (100–400 micrograms, depending on the local formulation used as standard-of-care at sites)) [20].

### Study procedures

Children were managed on general pediatric wards; ventilatory facilities were unavailable. Basic infrastructural support for emergency care, patient monitors, bedside hemoglobin, glucose and lactate point-of-care tests were provided. Local blood transfusion services provided blood free of charge, pre-screened for transfusion-transmissible infections and prepared using standard procedures, but without leucocyte reduction. Second transfusions, if indicated in immediate-transfusion participants, followed the originally-assigned randomized volume. Children in the control group who developed de novo severity criteria (hemoglobin < 4 g/dL or severity signs above) were given 20 ml/kg whole-blood equivalent. Furosemide or other diuretics were not prescribed. Other treatments, including anti-malarials and antibiotics, followed national guidelines. For ethical reasons screening and enrolment into the trial was halted when there were no donor blood units were available from the blood banks.

### Data collection

A structured case report form was completed at screening and at reviews every 30 min during transfusion, then regularly during admission. Haemoglobin was assessed using Hb 301 system (Hemocue® AB, Angelholm, Sweden) 8-hourly in the first 24 h and daily thereafter. After discharge, children were actively followed up on days 28, 90 and 180 post-randomisation for review of clinical status and haemoglobin measurements. At all follow up visits (including additional visits for illness) a record of was made of any hospitalisations and at Day 28 and Day 90 the carer brought back any unused tablets/sachets and these were recorded on a dedicated drug adherence proforma. Most readmissions (hospitalisations) were to the study hospitals where trial clinicians conducted a structured clinical assessment including basic diagnostic work-up. Serious adverse events (SAE), including re-admissions to the original or other hospitals, were actively solicited at every assessment.

The ethics committees of Imperial College London (ICREC 13–1-11), Makerere University, Uganda (SOMREC REF 2013–050), and the College of Medicine, Blantyre, Malawi (COMREC P.03/13/1365), approved the protocol. Where prior written consent from parents/legal guardians could not be obtained, ethics committees approved verbal assent with delayed written informed consent as soon as practicable [21].

### Outcomes

The primary outcome for this study was time from hospital discharge to first readmission within the 180 day follow up period. The incidence was estimated and predictors identified treating death before readmission as a competing risk. Children were censored at the earliest of date last known alive, or day 180 (6 months) from randomisation. Secondary outcomes for these analyses were time from discharge to first readmission for specific causes (anaemia, malaria (malaria film or RDT positive), or haemoglobinuria/dark urine syndrome (DUS)) [22]. As children could be re-admitted for multiple reasons over the 6-months of follow-up, and individual readmissions could also be for multiple reasons, when estimating incidence of readmission for each specific cause, we treated death without readmission for that cause as a competing risk, and any intervening admissions for other causes were ignored.

### Candidate predictors

All models adjusted for site (to control for minor differences in admission practices and management outside of the protocol), uncomplicated vs complicated severe anaemia (determining whether or not children could be randomised to no immediate transfusion) and whether or not the child received a transfusion in the primary admission. Other candidate predictors (listed in Supplementary Table 1) were from physical examination and assessment of severity at screening, clinical history of this illness, treatment in this illness, past history before this illness, and details of the child’s family. Results from the full blood count done at screening, transfusion blood pack type and age of donor blood were also included, as were the TRACT randomisations. Sickle cell disease status (HbSS (sickle cell disease, SCD) or HbAS (sickle cell trait) was obtained by batch genotyping stored admission samples at the end of the trial. We subclassified the HbSS genotype according to whether the child was known to have SCD at discharge (children reported to have SCD on admission, and 7 children found to have SCD during their first admission). Binary variables representing time between admission and randomisation (< 24 h vs > 24 h) and treatment adherence (missed one or more doses of MVMM, iron folate or cotrimoxazole by 28 days) were also included. A previous study found adherence had a beneficial effect on HIV survival in children even among those treated with placebo; therefore we have used this variable as a proxy for quality of care at home [23]. All data for the candidate predictors related to the primary admission.

### Statistical analysis of predictors of readmission

For the primary outcome, subhazard ratios for risk of readmission were estimated using a multivariable competing risks regression model, fitted using backwards stepwise elimination on complete cases for those variables in Supplementary Table 1 with < 5% missing data (including 2690 (69%) of 3894 children discharged alive from their primary admission). Continuous variables were modelled as fractional polynomials to allow for non-linearity using Stata mfp (alpha = 0.05). The model was refitted on cases that were complete on initially selected variables, and then other excluded variables (including those with > 5% missing data) were incorporated one-by-one. All additional factors with *p* < 0.1 were included in a second backwards elimination, retaining all originally selected factors regardless of significance. Interactions between the final included variables were explored, and backwards elimination performed on all interactions with *p* < 0.01. Variables with *p* < 0.05 in the final model were considered significant.

For each secondary outcome, a competing risks model included all variables from the all-cause model, then considered the other candidate variables as above. Finally models containing all factors identified as predictors of malaria or anaemia admissions were fitted together and a test of heterogeneity of effect on these two different causes was performed for each factor using stacked regression [24].

### Risk score for readmission

A risk score was developed based on the final all-cause readmission model as a mechanism to assess its overall predictive ability. Each included factor was assigned an integer value based on its hazard ratio, which was added to the total score if that factor was present. A score of 1 was assigned to factors with a hazard ratio of approximately 1.3–1.5. Continuous variables were dichotomised at a threshold approximating the same effect. Internal validity was assessed using the area under the receiver operating characteristic curve (AUROC) excluding deaths, since these could not be predicted by the competing risks model.

### “Phenotypes” of children at risk for readmission

To investigate groups of predictors that co-occurred in individual children presenting with severe anaemia, a hierarchical cluster analysis was performed using Ward’s linkage [25]. Clinical predictors in any of the all-cause or cause-specific models with < 10% missing data were included, with continuous factors rescaled so that all had equal variance. As the focus was on clinical factors, variables relating to the trial process, such as site, randomisation and blood pack, were not included. Strata (TRACT A or TRACT B- see above) was also excluded. However, respiratory distress and impaired consciousness (used to define anaemia severity) were added. The Duda Hart stopping rule [26] was used to determine the number of clusters, and comparisons of characteristics between clusters was performed using chi-squared tests or ANOVA for categorical and continuous factors, respectively.

## Results

### Incidence of readmission

From 17 September 2014 to 15 May 2017, 3983 eligible children were randomised in TRACT. 89 (2%) died in hospital, leaving 3894 (98%) children discharged alive and included in these analyses.

**Fig. 1:**
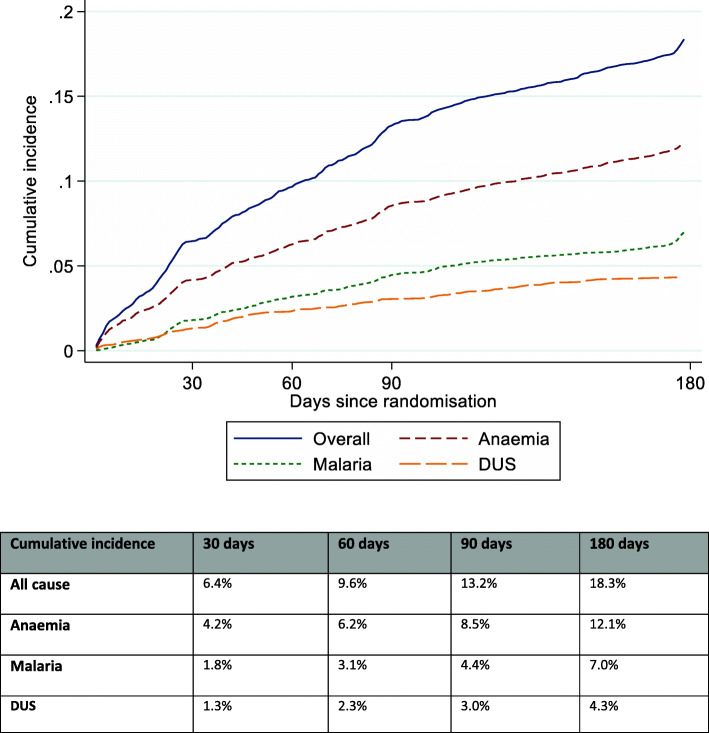
Cumulative incidence of readmissions from different causes (treating death as a competing risk)

Overall, 682 (18%) of these 3894 children were readmitted during follow-up to 180 days, for a total of 921 admissions (Table 1). Four hundred and three (10%) children were re-admitted on two or more separate occasions. The cumulative incidence of readmission (treating death as a competing risk) was 6.4% at 30 days post-discharge; 9.6% at 60 days; 13.2% at 90 days; and 18.3% at 180 days. The most common reasons for readmission were anaemia, malaria, other infections, and DUS/haemoglobinuria (Table 1; each admission could be for multiple reasons). In total, 456 (12%) children were readmitted for anaemia, 252 for malaria (6%) and 165 (4%) for DUS. The cumulative incidences at 90 and 180 days respectively were 8.5 and 12.1% for anaemia; 4.4 and 7.0% for malaria and 3.0 and 4.3% for DUS (Fig. 1).

**Table 1:** Causes of readmission post initial admission for severe anaemia

	Number (% of all readmissions)				
	Blantyre	Mulago	Soroti	Mbale	Total
**Total readmissions**	**79 (100%)**	**174 (100%)**	**281 (100%)**	**387 (100%)**	**921 (100%)**
**Anaemia readmissions**	**55 (70%)**	**89 (51%)**	**165 (59%)**	**300 (78%)**	**609 (66%)**
**Haemoglobinuria/DUS readmissions**	**0 (0%)**	**8 (5%)**	**87 (31%)**	**125 (32%)**	**220 (24%)**
**Malaria readmissions**	**28 (35%)**	**31 (18%)**	**94 (33%)**	**129 (33%)**	**282 (31%)**
**Other infection readmissions:**	**20 (25%)**	**64 (37%)**	**26 (9%)**	**181 (47%)**	**291 (32%)**
Sepsis	13 (16%)	29 (17%)	1 (0%)	153 (40%)	196 (21%)
Non-specific infection (fever)	0 (0%)	3 (2%)	0 (0%)	4 (1%)	7 (1%)
Respiratory	6 (8%)	20 (11%)	17 (6%)	32 (8%)	75 (8%)
Tuberculosis	0 (0%)	2 (1%)	0 (0%)	1 (0%)	3 (0%)
Gastrointestinal	1 (1%)	12 (7%)	3 (1%)	13 (3%)	29 (3%)
Bone infection	0 (0%)	1 (1%)	3 (1%)	1 (0%)	5 (1%)
Other	0 (0%)	5 (3%)	5 (2%)	1 (0%)	11 (1%)
**Other non-infection readmissions:**	**10 (13%)**	**56 (32%)**	**39 (14%)**	**29 (7%)**	**134 (15%)**
Sickle cell crisis	2 (3%)	24 (14%)	35 (12%)	11 (3%)	72 (8%)
Malignancy	7 (9%)	4 (2%)	2 (1%)	3 (1%)	16 (2%)
CNS	0 (0%)	2 (1%)	4 (1%)	2 (1%)	8 (1%)
Other	1 (1%)	27 (16%)	1 (0%)	11 (3%)	40 (4%)

Note: each readmission could have multiple causes and children could have multiple readmissions

There were 53/455 (12%) children with readmissions in Blantyre, 136/912 (15%) in Mulago, 215/1046 (21%) in Soroti and 278/1481 (19%) in Mbale

### Predictors of all-cause readmission using multivariable regression

A variety of clinical factors at the start of the primary admission for severe anaemia, underlying conditions and factors relating to management of previous illness were independently predictive of mortality (Table 2). The model included 3384/3894 (87%) of children discharged alive from their primary admission. Risk of readmission increased with younger age (subhazard ratio (SHR) 1.07 (95% confidence interval 1.03–1.10) per 1 year younger, *p* < 0.001) and was 2.5-fold higher in the 3% of children who were HIV positive (SHR 2.48 (1.63–3.78), *p* < 0.001). In contrast, children with known sickle cell disease had a 38% lower risk of readmission (SHR 0.62 (0.48–0.82), *p* = 0.01), with no evidence of variation in risk across the other SCD categories. Risk was also greater among children with two or more hospital admissions in the past year (SHR 1.44 (1.19–1.74), *p* < 0.001) and those who had received a blood transfusion prior to this illness (SHR 1.48 (1.13–1.93), *p* = 0.005). Presenting with chest indrawing at admission (SHR 1.36 (1.09–1.71), *p* = 0.01) or splenomegaly (SHR 1.26 (1.06–1.50) for enlarged, 1.47 (1.10–1.97) for gross, *p* = 0.01) also led to a greater risk of readmission, while children presenting with diarrhoea had a lower risk (SHR 0.71 (0.54–0.93), *p* = 0.01). Children admitted with malaria (positive rapid diagnostic test (RDT) or blood slide) who had not had a blood transfusion before this illness had a lower risk of readmission (SHR 0.60 (0.47–0.76), *p* < 0.001) whereas those with malaria who had been transfused before had a higher risk of readmission (SHR 1.58 (1.22–2.04); heterogeneity *p* < 0.001). Risk was also higher in children who had a longer stay in hospital in their primary admission (SHR 1.03 (1.00–1.06) per day longer, *p* = 0.04). Finally, having missed one or more doses of MVMM, iron folate or cotrimoxazole by the 28 day follow up visit (considered as a proxy for quality of care at home) was also associated with an increased risk of readmission (SHR 1.43 (1.21–1.69, *p* < 0.001) (Table 2). Of note, other factors did not add predictive power to this model, in particular there was no evidence of independent effects of last haemoglobin level recorded in the primary admission (SHR 0.98 (0.94–1.03) per g/dL higher, *p* = 0.47); nutritional status (SHR 1.01 (0.94–1.07) per 1 z-score higher weight-for-age, *p* = 0.85); or transfused blood pack type (SHR 0.85 (0.70–1.03) for red cell concentrates vs whole blood, *p* = 0.10).

**Table 2:** Predictors of readmission

Predictor	Readmitted (***N*** = 682, col % or median (IQR))	Not readmitted (***N*** = 3212, col % or median (IQR))	Univariate SHR (95% CI)	Multivariate SHR (95% CI)	***p*** value
Site^a^ - Mbale	278 (41%)	1203 (37%)	1.00	1.00	0.25
Blantyre	53 (8%)	402 (13%)	0.61 (0.45–0.82)	0.80 (0.57–1.12)	
Mulago	136 (20%)	776 (24%)	0.79 (0.65–0.97)	0.82 (0.65–1.03)	
Soroti	215 (32%)	831 (26%)	1.08 (0.90–1.29)	0.97 (0.79–1.19)	
Age at primary admission (per year younger)^a^	38.0 (17.0, 60.0)	34.0 (17.0, 62.0)	1.01 (0.98–1.03)	1.07 (1.03–1.10)	< 0.001
HIV positive^a^	26 (4%)	81 (3%)	1.52 (1.02–2.28)	2.48 (1.63–3.78)	< 0.001
Sickle status - AA	458 (67%)	2243 (70%)	1.00	1.00	0.01
AS	25 (4%)	99 (3%)	1.23 (0.82–1.84)	1.16 (0.75–1.81)	
SS, unknown at discharge	122 (18%)	486 (15%)	1.15 (0.94–1.39)	0.95 (0.76–1.20)	
SS, known at discharge	73 (11%)	353 (11%)	0.98 (0.77–1.25)	0.62 (0.46–0.82)	
Two or more hospital admissions in the last year before primary admission^a^	250 (37%)	651 (20%)	2.05 (1.76–2.40)	1.44 (1.19–1.74)	< 0.001
Received blood transfusion ever, prior to this illness^a^	341 (50%)	939 (29%)	2.17 (1.87–2.52)	1.48 (1.13–1.93)	0.005
History of cough at primary admission^a^	459 (67%)	1973 (61%)	1.27 (1.08–1.49)	1.13 (0.95–1.35)	0.16
Indrawing on admission^a^	118 (17%)	378 (12%)	1.51 (1.24–1.84)	1.36 (1.09–1.71)	0.01
Splenomegaly on admission^a^ - Not palpable	363 (53%)	2132 (66%)	1.00	1.00	0.01
Enlarged	248 (36%)	884 (28%)	1.57 (1.33–1.84)	1.26 (1.06–1.50)	
Gross	71 (10%)	190 (6%)	2.02 (1.57–2.61)	1.47 (1.10–1.97)	
Diarrhoea on admission	68 (10%)	437 (14%)	0.72 (0.56–0.92)	0.71 (0.54–0.93)	0.01
Received oral antimalarials in last week before primary admission	359 (53%)	1529 (48%)	1.21 (1.04–1.41)	1.08 (0.92–1.27)	0.36
Malaria positive at primary admission, no previous blood transfusion	195 (29%)	1533 (48%)	0.65 (0.52–0.81)	0.60 (0.47–0.76)	< 0.001
Malaria positive at primary admission, previous blood transfusion	209 (31%)	539 (17%)	1.73 (1.40–2.14)	1.58 (1.22–2.04)	< 0.001
Randomised > 24 h after admission^a^	37 (5%)	131 (4%)	1.28 (0.92–1.77)	1.36 (0.96–1.93)	0.08
Strata^a^ - TRACT A	452 (66%)	1884 (59%)	1.00	1.00	0.14
TRACT B, immediate transfusion	122 (18%)	655 (20%)	0.78 (0.64–0.95)	0.93 (0.74–1.17)	
TRACT B, triggered transfusion	69 (10%)	312 (10%)	0.91 (0.71–1.17)	1.05 (0.79–1.40)	
TRACT B, no transfusion	39 (6%)	361 (11%)	0.48 (0.35–0.67)	0.67 (0.47–0.96)	
Blood pack age (per week older)^a^	12.0 (7.0, 20.0)	12.0 (7.0, 17.0)	1.10 (1.03–1.18)	1.07 (0.99–1.15)	0.08
Length of stay (per day longer)^a^	3.0 (2.0, 5.0)	3.0 (2.0, 4.0)	1.05 (1.03–1.07)	1.03 (1.00–1.06)	0.04
Missed dose of MVMM, iron folate or cotrimoxazole by 28 days	245 (36%)	925 (29%)	1.35 (1.15–1.58)	1.43 (1.21–1.69)	< 0.001

^a^selected by initial backwards elimination: retained in final model regardless of significance for control of confounding

Note: Denominators based on children with observed data; numbers missing are given in Supplementary Table 1. Site and receipt of transfusion were included in all models. Final model included 3384/3894 (87%) children discharged alive and 609/682 (89%) readmitted children (exclusions due to missing data). All continuous factors were selected as linear. SHR = sub-hazard ratio from competing risks model (treating death before readmission as a competing risk). TRACT A = severe complicated anaemia (randomised to 30 vs 20 mls/kg transfusion). TRACT B = severe uncomplicated anaemia (randomised to immediate transfusion vs triggered transfusion; those who ultimately received a transfusion are labelled “triggered transfusion” and those who did not “no transfusion”)

### Predictors of anaemia, malaria and DUS readmissions using multivariable regression

Factors found to significantly increase the risk of readmission for anaemia in addition to the all-cause model were a lower haemoglobin at screening in their primary admission (1.16 (1.05–1.29) per g/dL lower, *p* = 0.01) (Table 3). For malaria readmissions, risk of readmission was doubled in children from a rural homestead (SHR 2.19 (0.99–4.84), *p* = 0.002) and children with a temperature gradient at screening in their primary admission (SHR 2.07 (1.28–3.35), *p* = 0.003). Children who were randomised to receive cotrimoxazole had a reduced risk of malaria readmission (SHR 0.75 (0.57–1.00), *p* = 0.05). Other factors which reduced risk were a higher monocyte count and receiving red cell concentrates rather than a whole blood transfusion, while risk increased among children who could walk unaided prior to admission (suggesting ambulant ‘older’ children are at more risk of mosquito/malaria exposure) and those with a higher platelet count (Table 3). For readmission with DUS, risk was higher among children with haemoglobinuria in their primary admission (SHR 2.26 (1.37–3.71), *p* = 0.001), those with a positive blood culture (SHR 3.25 (1.42–7.46), *p* = 0.01), and a higher granulocyte count at screening for their primary admission (SHR 1.35 (1.09–1.66) per 10 × 10^9^/L) (all *p* ≤ 0.01) (Table 3).

**Table 3:** Predictors of readmission for specified causes

Predictor	Anaemia SHR (95% CI)	***p***	Malaria SHR (95% CI)	***p***	Heterogeneity of effect between anaemia and malaria	DUS SHR (95% CI)	***p*** value
Site* - Mbale	1.00	< 0.001	1.00	0.99	0.09	1.00	< 0.001
Blantyre	0.68 (0.44–1.04)		0.91 (0.44–1.85)				
Mulago	0.56 (0.42–0.75)		0.95 (0.57–1.56)			0.14 (0.06–0.34)**	
Soroti	0.68 (0.53–0.88)		1.01 (0.69–1.48)			0.91 (0.57–1.47)	
Age at primary admission (per year younger)*	1.03 (0.99–1.07)	0.12	1.15 (1.07–1.23)	< 0.001	0.003	0.98 (0.91–1.06)	0.69
HIV positive*	2.48 (1.52–4.04)	< 0.001	0.85 (0.26–2.76)	0.79	0.05	–	–
Sickle status* - AA	1.00	< 0.001	1.00	0.24	0.48	1.00	0.01
AS	1.28 (0.77–2.14)		0.84 (0.34–2.05)			1.18 (0.45–3.11)	
SS, unknown at discharge	0.95 (0.72–1.26)		0.77 (0.44–1.36)			0.06 (0.01–0.41)	
SS, known at discharge	0.43 (0.30–0.64)		0.51 (0.26–1.00)			0.43 (0.20–0.95)	
Two or more hospital admissions in the last year before primary admission*	1.67 (1.33–2.11)	< 0.001	1.24 (0.88–1.74)	0.22	0.31	2.52 (1.63–3.91)	< 0.001
Received blood transfusion ever, prior to this illness*	1.67 (1.21–2.32)	0.002	2.19 (1.19–4.05)	0.01	0.50	2.74 (0.94–7.95)	0.06
History of cough at primary admission*	1.07 (0.87–1.33)	0.51	1.12 (0.82–1.52)	0.49	0.94	1.21 (0.80–1.83)	0.36
Indrawing on primary admission*	1.09 (0.82–1.44)	0.57	1.27 (0.83–1.94)	0.28	0.37	1.16 (0.66–2.03)	0.60
Splenomegaly on admission* - Not palpable	1.00	< 0.001	1.00	0.57	0.14	1.00	0.43
Enlarged	1.46 (1.18–1.82)		1.19 (0.87–1.63)			1.21 (0.76–1.94)	
Gross	1.73 (1.23–2.44)		1.14 (0.64–2.04)			0.82 (0.40–1.71)	
Diarrhoea on primary admission	0.73 (0.52–1.02)	0.07	0.72 (0.42–1.22)	0.22	0.79	0.57 (0.27–1.20)	0.14
Received oral antimalarials in last week before primary admission*	1.25 (1.02–1.53)	0.03	0.95 (0.71–1.27)	0.71	0.04	1.19 (0.82–1.73)	0.36
Malaria positive at primary admission, no previous blood transfusion	0.56 (0.41–0.77)	< 0.001	1.33 (0.77–2.32)	0.31	0.002	1.71 (0.66–4.43)	0.27
Malaria positive at primary admission, previous blood transfusion	1.52 (1.10–2.09)	0.02	2.61 (1.50–4.53)	0.74	0.07	4.48 (1.70–11.77)	0.94
Randomised > 24 h after admission*	1.49 (0.97–2.29)	0.07	0.91 (0.46–1.81)	0.79	0.09	1.70 (0.83–3.45)	0.14
Strata* - TRACT A	1.00	0.29	1.00	0.08	0.003	1.00	0.47
TRACT B, immediate transfusion	0.88 (0.64–1.21)		1.14 (0.76–1.71)			1.39 (0.80–2.41)	
TRACT B, triggered transfusion	1.12 (0.78–1.63)		1.59 (0.97–2.59)			1.31 (0.64–2.65)	
TRACT B, no transfusion	0.68 (0.41–1.11)		0.66 (0.33–1.31)				
Blood pack age (per week older)*	1.09 (1.00–1.18)	0.05	1.09 (0.96–1.24)	0.18	0.81	0.98 (0.83–1.15)	0.81
Length of stay (per day longer)	1.02 (0.98–1.05)	0.38	1.02 (0.97–1.08)	0.41	0.76	1.02 (0.95–1.10)	0.60
Missed dose of MVMM, iron folate or cotrimoxazole by 28 days*	1.34 (1.09–1.64)	0.005	1.25 (0.92–1.69)	0.16	0.34	1.67 (1.16–2.40)	0.01
Admitted > 24 h into another hospital at primary admission	0.74 (0.55–1.01)	0.06			0.74		
Haemoglobin at admission (per g/dL lower)	1.16 (1.05–1.29	0.01			0.83		
Type of homestead - Urban			1.00	0.002	0.003^1^		
Semi urban			0.46 (0.15–1.42)				
Rural			2.19 (0.99–4.84)				
Able to walk unaided before this illness at primary admission			1.79 (1.01–3.20)	0.05	0.04^2^		
Temperature gradient at primary admission			2.07 (1.28–3.35)	0.003	0.003^3^		
Cotrimoxazole randomisation- cotrimoxazole (vs no cotrimoxazole)			0.75 (0.57–1.00)	0.05	0.04^4^		
Monocytes (per 10^9^/L) at admission			0.87 (0.77–0.99)	0.03	0.03^5^		
Platelets (per 100 × 10^9^/L) at admission			1.10 (1.02–1.20)	0.02	0.11		
Blood pack type- settled (vs whole)			0.70 (0.49–1.00)	0.05	0.07		
Haemoglobinuria in this illness at primary admission						2.26 (1.37–3.71)	0.001
Liver > 2 cm below costal margin						1.47 (0.92–2.38)	0.11
Positive blood culture at primary admission						3.25 (1.42–7.46)	0.01
Granulocytes (per 10 × 10^9^/L) on primary admission						1.35 (1.09–1.66)	0.01

*selected by initial backwards elimination: retained in final model regardless of significance for control of confounding

** Blantyre and Mulago combined as there were no DUS readmissions in Blantyre

Note: Site and receipt of transfusion are included in all models. Anaemia: including 3381/3894 (87%) children discharged alive and 407/456 (89%) readmitted children (exclusions due to missing data). Malaria: including 3317/3894 (85%) children discharged alive, and 223/252 (88%) readmitted children. DUS: including 2633/3894 (68%) children discharged alive and 121/165 (73%) readmitted children. SHR = sub-hazard ratio from competing risks model (treating death before readmission for the specific cause as a competing risk). TRACT A = severe complicated anaemia (randomised to 30 vs 20 mls/kg transfusion). TRACT B = severe uncomplicated anaemia (randomised to immediate transfusion vs triggered transfusion; those who ultimately received a transfusion are labelled “triggered transfusion” and those who did not “no transfusion”)

HRs for anaemia readmission from stacked model:

^1^Type of homestead: semi urban 0.86 (0.53–1.41); rural 1.01 (0.66–1.54)

^2^Able to walk unaided before this illness at primary admission: 0.98 (0.68–1.42)

^3^Temperature gradient at primary admission: 1.04 (0.70–1.55)

^4^Cotrimoxazole randomisation- cotrimoxazole (vs no cotrimoxazole): 1.03 (0.83–1.27)

^5^Monocytes (per 10^9^/L) at admission: 1.02 (0.94–1.11)

The test for heterogeneity in effect between anaemia and malaria readmissions showed evidence of differences for several factors including age (*p* = 0.003); having had malaria at the primary admission (*p* = 0.002); trial strata and transfusion status (*p* = 0.003); and having received oral antimalarials in the week before primary admission (*p* = 0.04) (Table 3).

### Risk score for readmission

**Fig. 2:**
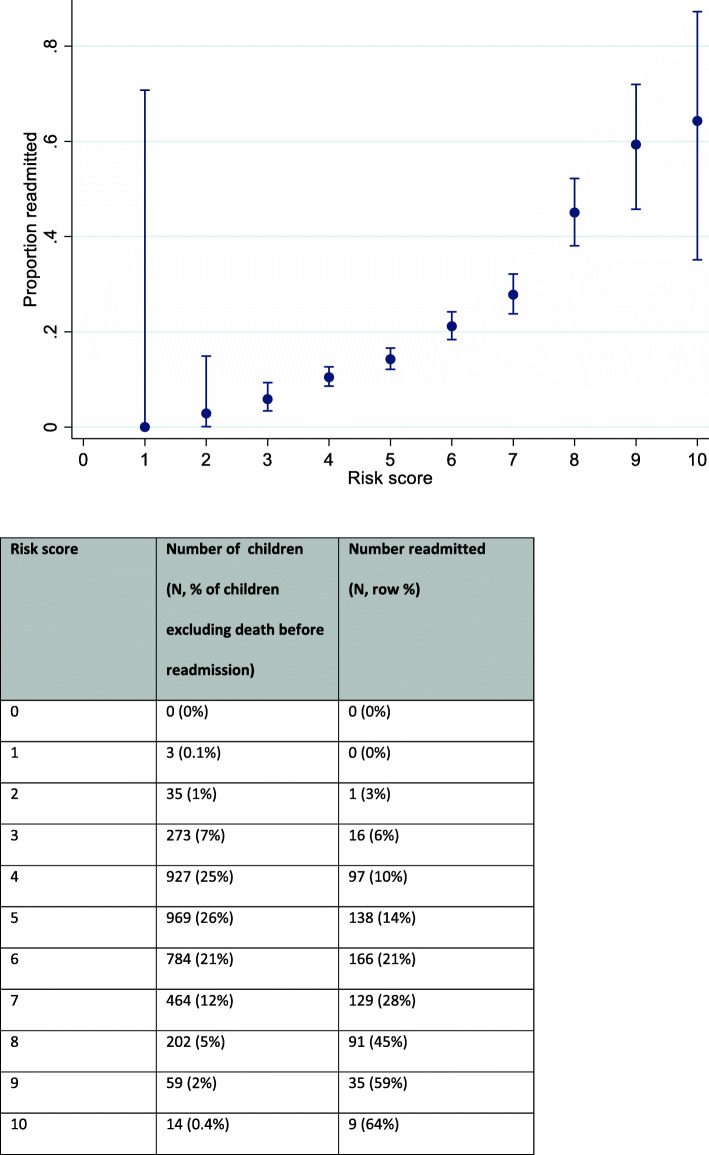
Proportion readmitted by risk score

The integer score reflecting the combined effect of the included factors on readmissions could theoretically range from 0 to 14 (Supplementary Table 2), although the maximum score observed in the data was 10. The probability of readmission increased steadily with increasing score (Fig. 2) with a 6% readmission rate for children with a score of 3 and a 45% readmission rate among those with a score of 8. However, the discriminatory ability of this score was relatively poor, with an AUROC of 0.68, excluding deaths.

### “Phenotypes” of children at risk for readmission

**Fig. 3:**
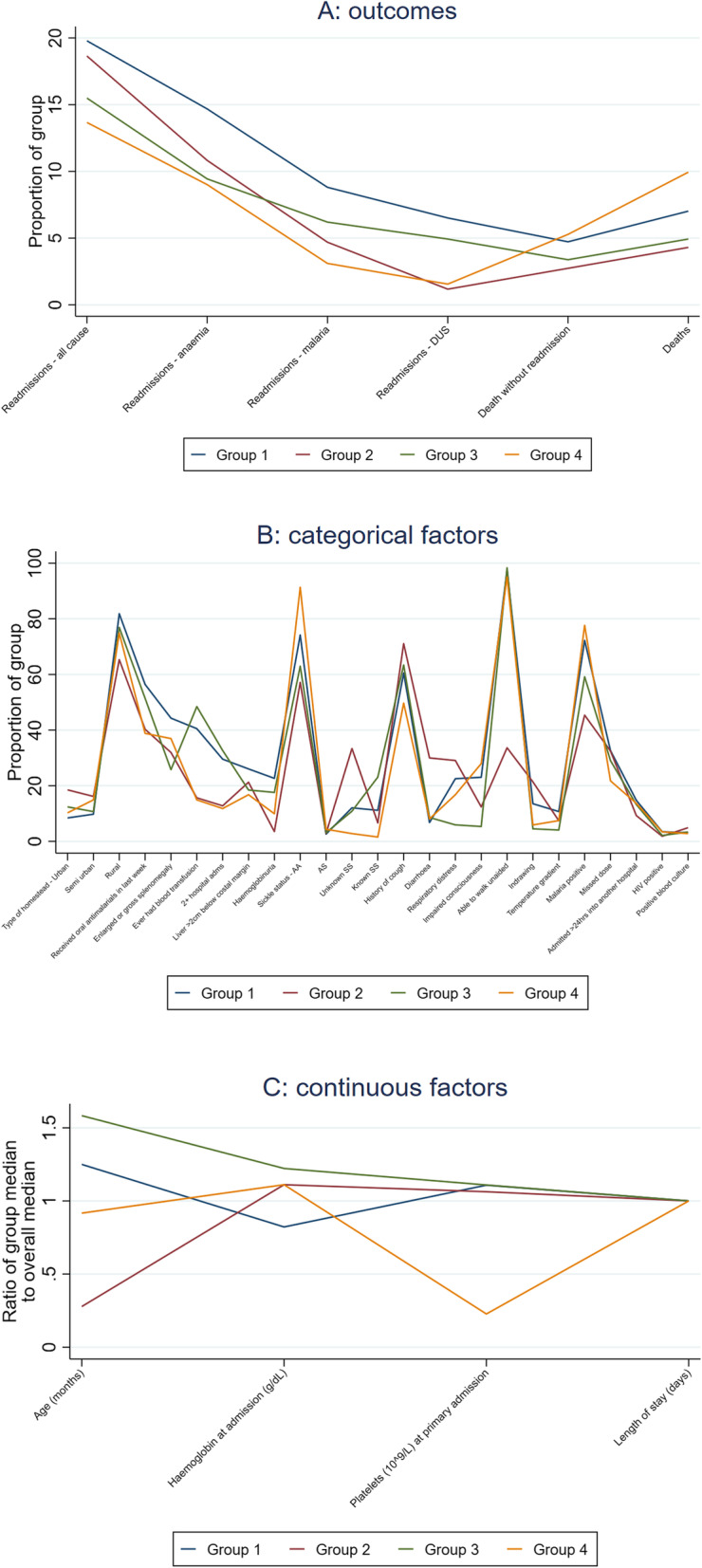
Cluster characteristics

As the underlying causes of severe anaemia are heterogeneous our cluster analysis sought to understand common factors that may co-occur in children at the time of the original admission that might predict readmission. Overall, four groups of children (“clusters”) were identified, comprising 47, 23, 21, and 10% of children with all-cause readmission rates 20, 19, 15, and 14% respectively (Supplementary Table 3). Details of cluster characteristics are presented in Fig. 3 and Supplementary Table 3. Most factors differed significantly across the four clusters (*p* ≤ 0.01), with the exception of HIV (*p* = 0.08) and positive blood culture (*p* = 0.16). Cluster 1 consisted of children with very low haemoglobin (mean 3.6 g/dL) at admission (with 60% having profound anaemia, Hb < 4 g/dL). A relatively high proportion had prior transfusions and previous hospital admissions, and 44% had enlarged spleen or gross splenomegaly, compared with 26–37% in other groups. Cluster 2 consisted of young children with a mean age of 10 months, a high proportion (33%) of whom had undiagnosed sickle cell disease. These children were more likely to present with diarrhoea (30%), and had low rates of haemoglobinuria (4%). Cluster 3 had the largest proportion of sickle-positive children (34%) and the highest previous healthcare usage (48% had a prior transfusion, and 33% had two or more hospital admissions in the past year). They were less likely than other groups to present with respiratory distress or impaired consciousness. Finally, cluster 4 consisted of children who were almost all sickle-negative (91%), but had higher rates of malaria (78%), and impaired consciousness (28%). They also had a low platelet count (mean 40 × 10^9^/L), consistent with a severe malaria phenotype [27]. This cluster had the lowest readmission rate (14%) but the highest proportion of deaths post-discharge (10%).

## Discussion

Overall, we found a high incidence of readmissions to hospital among Ugandan and Malawian children following an initial admission with severe anaemia. The readmission rate was highest in the first 30 days following randomisation, at 6%, dropping to approximately 3% per month thereafter; by 6-months 18% children had one or more readmissions. We aimed to investigate predictors of readmission to hospital and causes of readmissions. Although the retention to 6 months follow-up was excellent with very high ascertainment of vital status and re-admission events, limited tests available for diagnosing cause of readmissions meant we could only describe a few with any certainty. We found strong evidence for known predictors of increased risk of readmission, in particular, younger age and HIV infection. Unsurprisingly we also found a subgroup of children with repeated hospitalisations and/or prior transfusions who were at high risk of readmission. However, we did not identify any obvious clinical factors for intervention, with the possible exception of splenomegaly.

These results are broadly in agreement with a previous study looking at recurrence of severe anaemia in 377 children aged 6–60 months in Malawi [3]. Similar effects of younger age, HIV, malaria, and history of previous transfusion were found. In that study a history of jaundice was found to be strongly predictive of a recurrence of severe anaemia, but unlike our findings splenomegaly was not found to be have an independent effect.

With respect to factors related to blood transfusion (and health services) neither pack type (whole blood versus settled cells) nor the length of storage of donor blood packs had a significant effect on overall readmissions. We found for malaria re-admissions (7% of total readmissions) that there was a potentially lower risk of readmission with transfusion using red cell concentrates (adjusted odds ratio (AOR) 0.70 (0.49–1.00) *p* = 0.05). However, there was no association with pack-type in those readmitted with haemoglobinuria/dark water syndrome, a condition whose underlying aetiology strongly implicates active or recent malaria infection [22]; it therefore seems likely that this may be a chance finding. We had previously reported that neither whole-blood (44% of units transfused) or longer storage-age of donor blood adversely affected 28-day or 180-day mortality [19]. Thus, since pack type neither influenced the risk of mortality or readmission, component preparation, which has relatively recently been introduced to blood transfusion services in sub-Saharan Africa (sSA) at substantial cost, does not appear to be essential for safe paediatric transfusion practice. However, since all blood transfusions used in the TRACT trial were neither leucocyte-reduced, nor irradiated, as is standard practice in sSA [28], we were not able to establish whether this may be a risk factor for readmission (or any other transfusion-related immunomodulation (TRIM) adverse outcomes [29]).

Children with uncomplicated severe anaemia (Hb 4-6 g/dL without severity features) who never received a transfusion during the initial admission had 32% reduced risk of readmission. However, this sub-group may not have had the same baseline risk as the children randomised to no immediate transfusion who subsequently required a transfusion; therefore we cannot say that risk was reduced by not giving these children transfusions. Finally, the ‘missed dose’ factor representing adherence to randomised treatments, a putative proxy for quality of care, was highly predictive, suggesting that caregiver-related factors could also be important in driving readmissions [23].

Clinical factors identified related to severity (chest indrawing) and putatively malaria-endemicity (splenomegaly) were associated with increased risk of readmission to hospital. Gross splenomegaly (which we defined in our study proforma as 5 cm or more below the costal margin), was present in the original admission in 71 (10%) of the children who were readmitted and was found to be one factor which appeared to discriminate a subgroup at higher risk of readmission in cluster analysis. Children with a moderately enlarged spleen (present in 248 (36%) of readmitted children) also had increased risk of readmission compared to no palpable spleen found at the initial admission (1.26 (1.06–1.50)). Specifically, both the presence of gross splenomegaly or moderately enlarged spleen increased the risk by 1.73 (1.23–2.44) and 1.46 (1.18–1.82) respectively for anaemia readmissions. The presence of splenomegaly in a child admitted with severe anaemia could therefore be a potential target for future malaria prophylaxis intervention. Among children with gross splenomegaly at the original admission, 44% had HbSS (by genotype) compared to the prevalence of HbSS (26%) in those without moderate or no splenomegaly.

Splenomegaly is highly prevalent in children living in malarious areas [30], but has generally been considered as a relatively benign consequence of malaria exposure. Gross (chronic splenomegaly) on the other hand is less common and generally seen only in children exposed to high levels of malaria transmission [31]; and may incorporate a condition termed tropical splenomegaly syndrome or hyper-reactive malaria splenomegaly (HMS). HMS is believed to be the result of chronic antigenic stimulation derived from the malaria parasite and associated with elevated antimalarial antibodies [32]; genetic factors may contribute to its development. Although HMS is associated with poor long-term outcomes, there have been few recent clinical trials of short or longer term courses of antimalarial treatment/prophylaxis; most were small, were conducted 2–3 decades ago and focused on either chloroquine or proguanil [32]. The most recent Cochrane review of intermittent preventative antimalarial treatment (IPT) for children with severe anaemia [33] found no strong evidence that sulfadoxine-pyrimethamine [34] or lumefantrine artesunate [35] reduced readmissions/mortality in children hospitalised with severe anaemia; of note, the review did not specifically address the sub-group with splenomegaly.

The TRACT trial allowed us to test several hypotheses with regard to sickle cell genotype status since all children were genotyped and examined for four possible genotype status (normal, sickle cell trait (HbAS), known or unknown SCD). Whereas we might have expected undiagnosed SCD to increase risk of readmission and HbAS to reduce readmission compared to control, neither of these hypotheses were demonstrated. The latter can be explained as the numbers of children with HbAS were very few (only 4%), given background prevalence of sickle cell of 15%. As previously demonstrated, the substantially lower prevalence of HbAS in children with severe anaemia than the background suggest this is highly protective of hospitalisation with severe anaemia [36], owing to its high degree of malaria-specific resistance as when considering malaria and anaemia readmissions separately (Table 3; albeit not significant, potentially due to lack of power). DUS or blackwater fever, a typical triad of anaemia, passing of red or cola coloured urine and jaundice in the presence of recent or active malaria is a frequent cause for admission in Eastern Uganda [22]. In re-analysis of the FEAST trial data using a case-control design, we found no association between DUS and G6PD deficiency, but the known malaria protective genes (alpha thalassaemia and HbAS) were less common in DUS cases, providing further support for an aetiological role of malaria in DUS [22]. Even though culture-proven bacteraemia was uncommon (4%), the strong association of DUS readmission with a bacterial infection on the primary admission may provide some insights into the aetiological factors leading to recurrent DUS. Additional predictors of anaemia and malaria readmissions seemed to reflect intrinsic susceptibility to these conditions, as reflected in severity of initial presentation: for example, lower haemoglobin at the initial admission for anaemia readmission, and temperature gradient and rural homestead for malaria readmissions.

Recurrent admission to hospital, a “revolving door” syndrome, is not unique to children with severe anaemia. In a placebo-controlled trial of prophylactic cotrimoxazole in 1778 young (median age 11 months) Kenyan children hospitalised with severe and complicated malnutrition with HIV, in the year following enrolment there 227 (14.5%) deaths and 616 (35%) non-fatal hospital readmissions, with cotrimoxazole providing no benefit to either endpoint [37]. For Kenyan children discharged following an admission with severe pneumonia, the risk of post-discharge mortality (and thus possible readmission) was more than twice that of those admitted with other conditions [38], with any degree of undernutrition as the key risk factor in that study. While the authors suggested better micro- and macronutrient support could improve outcomes, this was not demonstrated in additional randomised comparisons (vitamin and macronutrient support) in TRACT [20]. Furthermore, in TRACT, although few children had severe malnutrition (*n* = 75), nutritional status was not a risk factor for readmission. A multicentre placebo-controlled trial of 3 days of dihydroartemisinin–piperaquine (DHA-PPQ) malaria chemoprevention administered at 2, 6, and 10 weeks post discharge to children admitted with severe anaemia and were followed for 180 days [39]. DHA-PPQ reduced readmissions by 70% (but not deaths) in the 3–14 weeks following discharge (i.e. up to 4 weeks following the last dose) but not beyond this. This may have future potential for reducing readmissions due to malaria in countries with seasonal malaria but less impact where malaria transmission is perennial.

As severe anaemia has multiple potential causes, the cluster analysis distilled this heterogeneous group into four clusters. Of these, SCD predominated in clusters 2 (chronic repeated admissions) and 3 (acute form, largely undiagnosed form). This further demonstrates the importance of screening all children admitted with severe anaemia for sickle cell disease, where it is endemic, since children with known SCD were less likely to be readmitted.

Limitations include possible omission of important clinical factors in this study, since most data were recorded at screening or admission. Detailed health status at discharge was not collected; for example, it is possible that haemoglobin measured at discharge would be a stronger predictor of readmission than haemoglobin at screening or 48 h (the latest timepoint tested consistently in all children). There were also limited data available on social/family circumstances, hence our use of “missed doses” of post-discharge medication as a proxy for quality of care from caregivers. There were also difficulties in assigning a predominant cause of readmission since each readmission could have multiple causes, with many children having combinations of malaria, anaemia, DUS and other infections which may have been less well investigated than during the primary admission. In addition, there were some differences in how conditions were described between sites and/or by clinical staff (Table 1). In particular, a high number of readmissions were attributed to sepsis; however, because one site never recorded this diagnosis, it was not considered reliable enough for cause-specific analysis. As our goal was to try to identify factors which could potentially be ameliorated by specific treatments that could be tested in future trials, we considered a large number of predictors (Supplementary Table 1), arguing that restricting to a smaller number based on current clinical opinion could miss important unknown potential predictors aspects. Ultimately, the low predictive power of our best-fitting model (AUROC only 0.68) suggests that the key determinants were not measured. Given this we did not validate the readmission score in an independent dataset.

## Conclusions

This study adds to the evidence on hospital readmission rates in children with severe anaemia and describes factors associated with increased risk of readmission for three major causes - anaemia, malaria and DUS.

Overall, we were not able to identify specific predictors of readmission that would be easily amenable to medical intervention. However, the fact that previous receipt of hospital services and carer adherence to medication were some of the strongest predictors, highlights the need for close follow up and support for parents with recurrently sick children needing admission to hospital.

## Supplementary Information

**Additional file 1: Supplementary Table 1.** Univariable summary statistics of all factors considered. **Supplementary Table 2.** Risk score calculation. **Supplementary Table 3.** Description of clusters.

## Data Availability

The data used in these analyses was collected as part of the TRACT trial, sponsored by Imperial College London, and is stored securely at MRC CTU at UCL. The datasets analysed during the current study are available from the corresponding author on reasonable request (r.connon@ucl.ac.uk).

## Abbreviations

AUROC: Area under the receiver operating curve
DUS: Dark urine syndrome
HMS: Hyper-reactive malaria splenomegaly
MVMM: Multivitamin multimineral supplements
SAE: Serious adverse events
SCD: Sickle cell disease
sSA: Sub-Saharan Africa
TRIM: Transfusion-related immunomodulation
