# Supplementary material for: Incidence and predictors of hospital readmission in children presenting with severe anaemia in Uganda and Malawi: a secondary analysis of TRACT trial data
Source: BMC Public Health. 2021 Jul 29;21:1480. doi: 10.1186/s12889-021-11481-6 (PMC8323322; doi:10.1186/s12889-021-11481-6)
Supplement: Supplementary file 1 — Additional file 1: Supplementary Table 1. Univariable summary statistics of all factors considered. Supplementary Table 2. Risk score calculation. Supplementary Table 3. Description of clusters. [file 12889_2021_11481_MOESM1_ESM.docx]

**Supplementary Table 1 Univariable summary statistics of all factors considered**

| **Candidate predictor** | **N (%) missing (N=3894)** | **Readmitted (N=682, col % or median (IQR))** | **Not readmitted (N=3212, col % or median (IQR))** | **SHR** | **95% CI** |
| --- | --- | --- | --- | --- | --- |
| Demographics at original admission | | | | | |
| Site - Mbale  Blantyre  Mulago  Soroti | 0 (0%) | 278 (41%)  53 (8%)  136 (20%)  215 (32%) | 1203 (37%)  402 (13%)  776 (24%)  831 (26%) | 1.00  0.61  0.79  1.08 | 0.45-0.82  0.65-0.97  0.90-1.29 |
| Age at admission (months, HR per year younger) | 0 (0%) | 38 (17, 60) | 34 (17, 62) | 1.01 | 0.98-1.03 |
| Sex (male vs female) | 0 (0%) | 390 (57%) | 1812 (56%) | 1.03 | 0.89-1.20 |
| Height for age z-score at admission (per unit higher) | 21 (1%) | -1.1 (-2.0, -0.1) | -1.2 (-2.1, -0.2) | 1.05 | 1.00-1.10 |
| Weight for age z-score at admission (per unit higher) | 112 (3%) | -1.3 (-2.1, -0.6) | -1.3 (-2.1, -0.6) | 1.03 | 0.97-1.09 |
| MUAC for age z-score at admission (per unit higher) | 14 (0%) | -1.0 (-1.8, -0.4) | -1.1 (-1.8, -0.4) | 1.02 | 0.96-1.09 |
| HIV positive | 191 (5%) | 26 (4%) | 81 (3%) | 1.52 | 1.02-2.28 |
| Sickle cell genotype- AA  AS  SS, unknown at discharge  SS, known at discharge | 35 (1%) | 458 (67%)  25 (4%)  122 (18%)  73 (11%) | 2243 (70%)  99 (3%)  486 (15%)  353 (11%) | 1.00  1.23  1.15  0.98 | 0.82-1.84  0.94-1.39  0.77-1.25 |
| Blood group- A  B  AB  O | 153 (4%) | 168 (25%)  166 (24%)  34 (5%)  296 (43%) | 847 (26%)  756 (24%)  164 (5%)  1310 (41%) | 1.00  1.10  1.04  1.13 | 0.88-1.36  0.72-1.51  0.93-1.36 |
| Two or more hospital admission in past year prior to primary admission | 36 (1%) | 250 (37%) | 651 (20%) | 2.05 | 1.76-2.40 |
| Received blood transfusion ever, prior to this illness | 31 (1%) | 341 (50%) | 939 (29%) | 2.17 | 1.87-2.52 |
| Able to walk without support before this illness | 74 (2%) | 558 (82%) | 2600 (81%) | 1.07 | 0.87-1.31 |
| Child's mother attended secondary school | 86 (2%) | 145 (21%) | 781 (24%) | 0.85 | 0.71-1.02 |
| Both parents still alive | 26 (1%) | 652 (96%) | 3068 (96%) | 0.90 | 0.62-1.31 |
| Child sleeps under bednet | 48 (1%) | 594 (87%) | 2680 (83%) | 1.27 | 1.01-1.61 |
| Type of homestead- Urban  Semi urban  Rural | 25 (1%) | 66 (10%)  59 (9%)  555 (81%) | 393 (12%)  398 (12%)  2398 (75%) | 1.00  0.89  1.31 | 0.63-1.27  1.02-1.70 |
| Number of siblings- 0  1  2  3  4 | 26 (1%)  26 (1%)  26 (1%)  26 (1%)  26 (1%) | 67 (10%)  104 (15%)  120 (18%)  126 (18%)  261 (38%) | 435 (14%)  594 (18%)  540 (17%)  475 (15%)  1146 (36%) | 1.00  1.13  1.40  1.63  1.43 | 0.83-1.53  1.04-1.89  1.21-2.19  1.09-1.86 |
| Vital signs at primary admission | | | | | |
| Heart rate (bpm) | 0 (0%) | 148 (133, 161) | 146 (130, 160) | 1.00 | 1.00-1.01 |
| Temperature (°C) | 0 (0%) | 37.3 (36.7, 38.0) | 37.3 (36.7, 37.9) | 1.04 | 0.96-1.12 |
| Systolic blood pressure (mmHg) | 1 (0%) | 92 (84, 99) | 91 (84, 99) | 1.00 | 0.99-1.01 |
| Diastolic blood pressure (mmHg) | 4 (0%) | 54.5 (46, 61) | 54 (48, 62) | 1.00 | 0.99-1.00 |
| Oxygen saturation | 2 (0%) | 97 (95, 99) | 98 (95, 99) | 0.98 | 0.96-1.00 |
| Respiratory rate | 0 (0%) | 41 (34, 50) | 40 (33, 49) | 1.01 | 1.00-1.01 |
| Received glucose | 5 (0%) | 27 (4%) | 72 (2%) | 1.65 | 1.14-2.41 |
| Received oxygen | 6 (0%) | 43 (6%) | 161 (5%) | 1.28 | 0.94-1.76 |
| Capillary refill time >2 seconds | 10 (0%) | 179 (26%) | 683 (21%) | 1.28 | 1.08-1.52 |
| Temperature gradient | 10 (0%) | 63 (9%) | 275 (9%) | 1.09 | 0.84-1.42 |
| Weak radial pulse volume | 20 (1%) | 17 (2%) | 75 (2%) | 1.10 | 0.68-1.78 |
| Clinical history of presenting illness at primary admission | | | | | |
| History of fever for more than 14 days | 21 (1%) | 33 (5%) | 155 (5%) | 1.05 | 0.73-1.49 |
| History of cough | 1 (0%) | 459 (67%) | 1973 (61%) | 1.27 | 1.08-1.49 |
| Increased work of breathing | 16 (0%) | 174 (26%) | 710 (22%) | 1.19 | 1.00-1.41 |
| Indrawing on admission | 5 (0%) | 118 (17%) | 378 (12%) | 1.51 | 1.24-1.84 |
| Vomiting | 6 (0%) | 415 (61%) | 1832 (57%) | 1.15 | 0.98-1.34 |
| Haemoglobinuria in this illness | 18 (0%) | 136 (20%) | 471 (15%) | 1.42 | 1.17-1.72 |
| Fits in this illness | 7 (0%) | 38 (6%) | 214 (7%) | 0.87 | 0.63-1.22 |
| Prostrate | 5 (0%) | 151 (22%) | 663 (21%) | 1.11 | 0.93-1.33 |
| Blantyre Coma Score (per increase of 1) | 12 (0%) | 5 (5, 5) | 5 (5, 5) | 0.90 | 0.81-1.01 |
| Deep breathing | 6 (0%) | 118 (17%) | 465 (14%) | 1.23 | 1.01-1.51 |
| Crackles | 32 (1%) | 60 (9%) | 194 (6%) | 1.44 | 1.10-1.87 |
| Sunken eyes | 2 (0%) | 48 (7%) | 166 (5%) | 1.33 | 0.99-1.77 |
| Cold hands | 3 (0%) | 30 (4%) | 86 (3%) | 1.59 | 1.10-2.28 |
| Liver >2cm below costal margin | 10 (0%) | 193 (28%) | 637 (20%) | 1.54 | 1.30-1.82 |
| Splenomegaly- Not palpable  Enlarged  Gross | 6 (0%) | 363 (53%)  248 (36%)  71 (10%) | 2132 (66%)  884 (28%)  190 (6%) | 1.00  1.57  2.02 | 1.33-1.84  1.57-2.61 |
| Diarrhoea on admission | 4 (0%) | 68 (10%) | 437 (14%) | 0.72 | 0.56-0.92 |
| Jaundice | 5 (0%) | 303 (44%) | 1026 (32%) | 1.61 | 1.38-1.87 |
| Kwashiorkor | 8 (0%) | 18 (3%) | 109 (3%) | 0.79 | 0.49-1.26 |
| Admitted for over 24 hours into another hospital | 3 (0%) | 87 (13%) | 413 (13%) | 0.99 | 0.79-1.23 |
| 2 or more doses of IV or IM quinine/artesunate | 91 (2%) | 135 (20%) | 652 (20%) | 0.95 | 0.79-1.15 |
| Received oral antimalarials in last week | 47 (1%) | 359 (53%) | 1529 (48%) | 1.21 | 1.04-1.41 |
| Received oral antibiotics in last week | 83 (2%) | 223 (33%) | 893 (28%) | 1.25 | 1.06-1.47 |
| Received traditional medicine in last week | 25 (1%) | 90 (13%) | 362 (11%) | 1.17 | 0.94-1.46 |
| Received antihelminths in last 6 months | 89 (2%) | 169 (25%) | 798 (25%) | 1.01 | 0.85-1.20 |
| Laboratory tests at primary admission | | | | | |
| Haemoglobin at screening (per 1g/dl lower) | 0 (0%) | 4.3 (3.4, 5.1) | 4.6 (3.7, 5.4) | 1.19 | 1.11-1.27 |
| Glucose at admission (mmol/L) | 32 (1%) | 5.6 (5.0, 6.3) | 5.6 (4.9, 6.4) | 0.98 | 0.92-1.04 |
| Lactate at admission (mmol/L) | 37 (1%) | 2.8 (1.9, 4.4) | 2.7 (1.8, 4.1) | 1.03 | 1.01-1.06 |
| Malaria (rapid diagnostic test or blood slide) | 7 (0%) | 404 (59%) | 2085 (65%) | 0.80 | 0.69-0.93 |
| Positive blood culture at screening | 526 (14%) | 21 (3%) | 100 (3%) | 0.99 | 0.64-1.52 |
| Received antibiotics on admission | 0 (0%) | 489 (72%) | 2178 (68%) | 1.18 | 1.00-1.40 |
| White blood cell count (per 10 x 10^9^/L) on admission | 146 (4%) | 15.1 (9.2, 26.9) | 14.9 (8.9, 25.1) | 1.00 | 1.00-1.01 |
| MCV at admission (per fL) | 126 (3%) | 80.0 (73.8, 86.8) | 79.1 (72.5, 86.4) | 1.01 | 1.00-1.01 |
| MCH at admission (per picogram) | 124 (3%) | 25.9 (23.6, 28.3) | 25.5 (23.1, 27.9) | 1.02 | 1.01-1.04 |
| MCHC at admission (per g/dL) | 133 (3%) | 31.9 (30.0, 33.8) | 31.9 (30.0, 33.7) | 1.01 | 0.99-1.04 |
| Platelets (per 10 x 10^9^/L) at admission | 134 (3%) | 174.0 (100.0, 300.0) | 176.0 (96.0, 302.0) | 1.00 | 1.00-1.00 |
| CRP (per 10 mg/L) at admission | 192 (5%) | 56.5 (18.5, 97.5) | 61.6 (23.6, 116.8) | 1.00 | 1.00-1.00 |
| Lymphocytes (per 10 x 10^9^/L) at admission | 576 (15%) | 5.7 (3.2, 10.5) | 5.7 (3.1, 10.4) | 0.98 | 0.89-1.08 |
| Granulocytes (per 10 x 10^9^/L) at admission | 604 (16%) | 6.6 (3.6, 11.4) | 5.9 (3.3, 10.6) | 1.03 | 0.94-1.14 |
| Monocytes (per 10^9^/L) at admission | 582 (15%) | 1.0 (0.5, 1.9) | 1.1 (0.6, 1.9) | 0.99 | 0.94-1.05 |
| Last recorded haemoglobin in admission (per g/dl) | 7 (0%) | 7.1 (5.7, 8.4) | 7.1 (5.9, 8.5) | 0.96 | 0.93-1.00 |
| Original hospitalisation | | | | | |
| Randomised > 24h after admission | 0 (0%) | 37 (5%) | 131 (4%) | 1.28 | 0.92-1.77 |
| TRACT A  TRACT B, immediate transfusion  TRACT B, deferred transfusion  TRACT B, no transfusion | 0 (0%) | 452 (66%)  122 (18%)  69 (10%)  39 (6%) | 1884 (59%)  655 (20%)  312 (10%)  361 (11%) | 1.00  0.78  0.91  0.48 | 0.64-0.95  0.71-1.17  0.35-0.67 |
| Blood pack age if transfused (days, HR per 7 days) | 19 (0%) | 12 (7, 20) | 12 (7, 17) | 1.10 | 1.03-1.18 |
| Blood pack type- settled (vs whole) (if transfused) | 403 (10%) | 346 (51%) | 1556 (48%) | 1.10 | 0.95-1.28 |
| Randomisation 1- immediate (vs triggered) | 2336 (60%) | 122 (18%) | 655 (20%) | 1.13 | 0.87-1.46 |
| Randomisation 1- 30mls (vs 20mls) | 781 (20%) | 297 (44%) | 1259 (39%) | 1.07 | 0.91-1.26 |
| Randomisation 2- MVMM (vs iron folate) | 0 (0%) | 335 (49%) | 1623 (51%) | 0.95 | 0.82-1.11 |
| Randomisation 3- Cotrimoxazole (vs no cotrimoxazole) | 0 (0%) | 332 (49%) | 1614 (50%) | 0.95 | 0.82-1.10 |
| Length of stay (per day) | 0 (0%) | 3 (2, 5) | 3 (2, 4) | 1.05 | 1.03-1.07 |
| Follow up | | | | | |
| Missed dose of MVMM, iron folate or cotrimoxazole by 28 day visit | 0 (0%) | 245 (36%) | 925 (29%) | 1.35 | 1.15-1.58 |

Note: TRACT A=severe complicated anaemia (randomised to 30 vs 20 mls/kg transfusion). TRACT B=severe uncomplicated anaemia (randomised to immediate transfusion vs triggered transfusion; those who ultimately received a transfusion are labelled “deferred transfusion” and those who did not “no transfusion”).

**Supplementary Table 2 Risk score calculation**

| **Predictor** | **Multivariate HR (95% CI)** | **Score value given** |
| --- | --- | --- |
| Site - Mbale  Blantyre  Mulago  Soroti | 1.00  0.80 (0.57-1.12)  0.82 (0.65-1.02)  0.97 (0.79-1.19) | 0  0  0  0 |
| Age at primary admission (per year younger) | 1.07 (1.03-1.10) | +1 if <7 years |
| HIV positive | 2.48 (1.63-3.77) | 2 |
| Sickle status - AA  AS  SS, unknown at discharge  SS, known at discharge | 1.00  1.17 (0.75-1.81)  0.97 (0.77-1.21)  0.60 (0.45-0.80) | 1  1  1  0 |
| Two or more hospital admissions in the last year before primary admission | 1.45 (1.20-1.75) | 1 |
| Received blood transfusion ever, prior to this illness | 1.48 (1.13-1.94) | 1 |
| History of cough at primary admission | 1.13 (0.95-1.35) | 0 |
| Indrawing on admission | 1.36 (1.09-1.71) | 1 |
| Diarrhoea on admission | 0.71 (0.54-0.93) | 1 |
| Splenomegaly on admission - Not palpable  Enlarged  Gross | 1.00  1.26 (1.06-1.50)  1.47 (1.10-1.97) | 0  0  1 |
| Received oral antimalarials in last week before primary admission | 1.08 (0.92-1.27) | 0 |
| Malaria positive at primary admission, no previous blood transfusion | 0.60 (0.47-0.77) | +1 if no malaria |
| Malaria positive at primary admission, previous blood transfusion | 1.58 (1.22-2.04) | 1 |
| Randomised >24h after admission | 1.36 (0.96-1.93) | 1 |
| Strata - TRACT A  TRACT B, immediate transfusion  TRACT B, deferred transfusion  TRACT B, no transfusion | 1.00  0.92 (0.74-1.16)  1.04 (0.78-1.38)  0.67 (0.47-0.96) | 1  1  1  0 |
| Blood pack age (per week older) | 1.07 (0.99-1.14) | +1 if >5 weeks |
| Length of stay (per day longer) | 1.03 (1.00-1.06) | +1 if >7 days |
| Missed dose of MVMM, iron folate or cotrimoxazole by 28 days | 1.43 (1.21-1.69) | 1 |

**Supplementary Table 3 Description of clusters**

| **Outcome** | **Cluster 1** | **Cluster 2** | **Cluster 3** | **Cluster 4** | **p** |
| --- | --- | --- | --- | --- | --- |
| N (row %) | 1567 (47%) | 767 (23%) | 710 (21%) | 322 (10%) | . |
| Readmissions - all cause | 310 (20%) | 143 (19%) | 110 (15%) | 44 (14%) | 0.01 |
| Readmissions - anaemia | 230 (15%) | 83 (11%) | 67 (9%) | 29 (9%) | <0.001 |
| Readmissions - malaria | 138 (9%) | 36 (5%) | 44 (6%) | 10 (3%) | <0.001 |
| Readmissions - DUS | 102 (7%) | 9 (1%) | 35 (5%) | 5 (2%) | <0.001 |
| Death without readmission | 74 (5%) | 21 (3%) | 24 (3%) | 17 (5%) | 0.06 |
| Deaths | 110 (7%) | 33 (4%) | 35 (5%) | 32 (10%) | 0.001 |
| Mean risk score | 5.6 | 5.1 | 5.3 | 4.8 | <0.001 |
| **Predictor** |  |  |  |  |  |
| Type of homestead - Urban  Semi urban  Rural | 8%  10%  82% | 19%  16%  65% | 12%  11%  77% | 10%  15%  75% | <0.001 |
| Age at primary admission (months) | 45 | 10 | 57 | 33 | <0.001 |
| HIV positive | 3% | 2% | 2% | 3% | 0.08 |
| Sickle status - AA  AS  SS, unknown at discharge  SS, known at discharge | 74%  3%  12%  11% | 57%  3%  33%  7% | 63%  3%  11%  23% | 91%  4%  3%  2% | <0.001 |
| Two or more hospital admissions in the last year before primary admission | 30% | 13% | 33% | 12% | <0.001 |
| Received blood transfusion ever, prior to this illness | 41% | 16% | 48% | 15% | <0.001 |
| Able to walk unaided before this illness at primary admission | 98% | 34% | 98% | 95% | <0.001 |
| Respiratory distress | 23% | 29% | 6% | 17% | <0.001 |
| Impaired consciousness | 23% | 12% | 5% | 28% | <0.001 |
| Temperature gradient at primary admission | 11% | 7% | 4% | 7% | <0.001 |
| History of cough at primary admission | 60% | 71% | 63% | 50% | <0.001 |
| Indrawing on admission | 14% | 21% | 5% | 6% | <0.001 |
| Diarrhoea on admission | 7% | 30% | 9% | 8% | <0.001 |
| Haemoglobinuria in this illness at primary admission | 23% | 4% | 18% | 10% | <0.001 |
| Liver >2cm below costal margin | 26% | 21% | 18% | 17% | <0.001 |
| Splenomegaly on admission - Not palpable  Enlarged  Gross | 56%  35%  9% | 68%  27%  5% | 74%  20%  6% | 63%  30%  7% | <0.001 |
| Admitted >24 hours into another hospital at primary admission | 15% | 9% | 13% | 14% | 0.003 |
| Received oral antimalarials in last week before primary admission | 56% | 40% | 51% | 39% | <0.001 |
| Haemoglobin at admission (g/dL) | 3.7 | 5.0 | 5.5 | 5.0 | <0.001 |
| Malaria positive at primary admission | 72% | 45% | 59% | 78% | <0.001 |
| Positive blood culture at primary admission | 3% | 5% | 3% | 3% | 0.16 |
| Platelets (10^9^/L) at primary admission | 195 | 187 | 195 | 40 | <0.001 |
| Length of stay (days) | 3 | 3 | 3 | 3 | 0.01 |
| Missed dose of MVMM, iron folate or cotrimoxazole by 28 days | 33% | 32% | 29% | 22% | <0.001 |

Note: all percentages are column percentages of total children in each Cluster, except where shown, for categorical data and means for continuous data.
